# The Use of Large Probes

**Published:** 1877-10-20

**Authors:** 


The use of Large Probes in the Treatment of Strictures of the Nasal Duct.—By Samuel Theobald, M. D., Surgeon to the Baltimore Charity Eye and Ear Dispensary; Opthalmic and Aural Surgeon to St. Vincent's Hospital, Baltimore.

